# Expert and Advocacy Group Consensus Findings on the Horizon of Public Health Genetic Testing

**DOI:** 10.3390/healthcare4010014

**Published:** 2016-01-27

**Authors:** Stephen M. Modell, Karen Greendale, Toby Citrin, Sharon L. R. Kardia

**Affiliations:** 1 Departments of Health Management and Policy, and Epidemiology, University of Michigan School of Public Health, 4628 SPH Tower, 1415 Washington Heights, Ann Arbor, MI 48109-2029, USA; 2 Department of Health Policy, Management and Behavior, School of Public Health, State University of New York at Albany, One University Place, Rm. 169, Rensselaer, NY 12144-3456, USA; karengreendale@gmail.com; 3 Department of Health Management and Policy, University of Michigan School of Public Health, M3049, SPH II, 1415 Washington Hts., Ann Arbor, MI 48109-2029, USA; tcitrin@umich.edu; 4 Department of Epidemiology, University of Michigan School of Public Health, 4619A SPH Tower, 1415 Washington Hts., Ann Arbor, MI 48109-2029, USA; skardia@umich.edu

**Keywords:** health policy, genomics, cascade screening, electronic health records, surveillance, hereditary breast and ovarian cancer, familial hypercholesterolemia, Lynch syndrome, consumer advocacy, consensus

## Abstract

Description: Among the two leading causes of death in the United States, each responsible for one in every four deaths, heart disease costs Americans $300 billion, while cancer costs Americans $216 billion per year. They also rank among the top three causes of death in Europe and Asia. In 2012 the University of Michigan Center for Public Health and Community Genomics and Genetic Alliance, with the support of the Centers for Disease Control and Prevention Office of Public Health Genomics, hosted a conference in Atlanta, Georgia to consider related action strategies based on public health genomics. The aim of the conference was consensus building on recommendations to implement genetic screening for three major heritable contributors to these mortality and cost figures: hereditary breast and ovarian cancer (HBOC), familial hypercholesterolemia (FH), and Lynch syndrome (LS). Genetic applications for these three conditions are labeled with a “Tier 1” designation by the U.S. Centers for Disease Control and Prevention because they have been fully validated and clinical practice guidelines based on systematic review support them. Methodology: The conference followed a deliberative sequence starting with nationally recognized clinical and public health presenters for each condition, followed by a Patient and Community Perspectives Panel, working group sessions for each of the conditions, and a final plenary session. The 74 conference participants represented disease research and advocacy, public health, medicine and nursing, genetics, governmental health agencies, and industry. Participants drew on a public health framework interconnecting policy, clinical intervention, surveillance, and educational functions for their deliberations. Results: Participants emphasized the importance of collaboration between clinical, public health, and advocacy groups in implementing Tier 1 genetic screening. Advocacy groups could help with individual and institutional buy-in of Tier 1 programs. Groups differed on funding strategies, with alternative options such as large-scale federal funding and smaller scale, incremental funding solutions proposed. Piggybacking on existing federal breast and colorectal cancer control programs was suggested. Public health departments need to assess what information is now being collected by their state cancer registries. The groups advised that information on cascade screening of relatives be included in toolkits for use by states. Participants stressed incorporation of family history into health department breast cancer screening programs, and clinical HBOC data into state surveillance systems. The carrying out of universal LS screening of tumors in those with colorectal cancer was reviewed. Expansion of universal screening to include endometrial tumors was discussed, as was the application of guidelines recommending cholesterol screening of children 9–11 years old. States more advanced in terms of Tier 1 testing could serve as models and partners with other states launching screening and surveillance programs. A multidisciplinary team of screening program champions was suggested as a means of raising awareness among the consumer and health care communities. Participants offered multiple recommendations regarding use of electronic health records, including flagging of at-risk family members and utilization of state-level health information exchanges. The paper contains an update of policy developments and happenings for all three Tier 1 conditions, as well as identified gaps. Conclusions: Implementation of cascade screening of family members for HBOC and FH, and universal screening for LS in CRC tumors has reached a point of readiness within the U.S., with creative solutions at hand. Facilitating factors such as screening coverage through the Patient Protection and Affordable Care Act, and state health information exchanges can be tapped. Collaboration is needed between public health departments, health care systems, disease advocacy groups, and industry to fully realize Tier 1 genetic screening. State health department and disease networks currently engaged in Tier 1 screening can serve as models for the launch of new initiatives.

## 1. Introduction: A Gathering to Focus on Winnable Battles in Public Health Genomics

When the term “public health genetics” is mentioned, what comes to mind are the hemoglobinopathy (sickle cell + thalassemia) and newborn screening programs launched in the 1970s. These programs have been established with lessons learned along the way. Where are we headed from here? We now use the term “public health genomics”, but what exactly does this term encompass? Certainly the Human Genome Project and progeny programs have revealed an array of genetic variants which impact the health of individuals and families. These discoveries have made possible genetic tests for numerous cancer conditions, and for certain cardiovascular diseases as well. Those in the field of Public Health Genomics envision a time when major common, chronic diseases with a significant genetic component can be assessed on a wider scale, with screening programs available to identify all those at high risk. It is important to keep in mind, however, that genomic applications are best initiated when a confluence of evidence and societal attitude indicate the time is ready for translation into the public domain [1].

In September 2012 a multi-sectoral group of clinical and public health experts and consumer advocates convened in Atlanta, Georgia to consider in a measured yet visionary way what steps might be taken to address and undertake “winnable battles” in public health genomics while avoiding the potential pitfalls associated with unvalidated genetic technology. This consensus building event was hosted by the United States (U.S.) Centers for Disease Control and Prevention—Office of Public Health Genomics (CDC-OPHG), and planned and executed by the University of Michigan Center for Public Health and Community Genomics and the non-profit Genetic Alliance. The diverse group of participants was asked to form recommendations concerning the implementation of testing for three major genetic conditions—hereditary breast and ovarian cancer (HBOC), Lynch syndrome (LS) or hereditary nonpolyposis colorectal cancer, and familial hypercholesterolemia (FH). This pursuit was a natural “next step” for the three organizations, which had recently collaborated on mapping a vision of public health genomics over the next five years. The *Proceedings* of the earlier meeting had recommended validated use of family health history and cascade screening to identify affected family members, and suggested that means should be taken, including the development of funding and data collection strategies, to further the utilization of such tools [2].

Concurrently, members of the public health community in the United States had been calling for practice to move from a unilateral focus on newborn screening and services for children with genetic diseases or birth defects into the chronic diseases arena [3]. The need is palpable given that heart disease and cancer are each responsible for one in four deaths in the U.S., and cost Americans $300 and $216 billion per year, respectively. They are also among the top three leading causes of death in Europe and Asia. The desire to prevent morbidity and mortality due to diseases manifesting at all ages contributed to the 2012 consensus conference event title—*New Strategies in Public Health Genomics: Actions to Save Lives Now*. These forward-projecting forces have been viewed through the lens of undertaking “winnable battles” aimed at leading causes of death and disability where measurable impacts can be achieved. Following a brief presentation by Ursula Bauer, Ph.D., M.P.H., director of the National Center for Chronic Disease Prevention and Health Promotion on modification of the major risk factors for the most common chronic diseases, conference participants were charged with addressing how to make a significant and measurable impact on morbidity and mortality due to HBOC, FH, and LS, for which “Tier 1” implementation strategies are available [4,5] (Table 1).

Cascade screening of relatives for FH via DNA or cholesterol testing, “snowballing” from one positively identified relative to another, is a “Tier 1” application for which the base of collected evidence on clinical validity and utility supports implementation into practice [5,6]. An example of a “Tier 2” application, where the existing evidence supports informed provider and consumer decision-making but is insufficient to justify routine implementation in practice, is UGT1A1 genotyping to identify patients at-risk for irinotecan toxicity in the treatment of metastatic colorectal cancer [7,8]. Genetic as opposed to conventional testing for hereditary hemochromatosis or iron overload disorder is an example of a “Tier 3” application; the synthesized evidence indicates that genetic testing is not ready for routine practice, but such testing may be considered in clinical and population research [9]. The stage is set for a systematic effort, utilizing empirical knowledge and consensus building, towards evaluating and promoting those categories of genetic testing which have been shown to have definitive rather than putative value for the health of populations. However, just as individual studies can have limited power which reduces the validity of their results, consensus building can lack power if adequate and informed representation is not assured. The genetic testing content area for the three select genomic applications which have achieved Tier 1 status will be covered from the perspective of 74 professionals and disease advocates taking part in the *New Strategies* conference [10]. Recommendations emerging from consensus building during the conference, which ranged from effective inter-organizational collaboration to modifications in practice and electronic storage of data and their relevance for current events in the field, will be described. These proposals need to be viewed in the context of the current fiscal environment, which remains conservative despite the advent of the Patient Protection and Affordable Care Act. Public health genomics applications will be vying with many other time-tested public health initiatives with which the public and health practitioners are comfortable. In addition to individuals being confident that Tier 1 genetic applications are valid and reliable, they must feel that they can be used as easily and readily as other interventions, and that a system is in place for their use [11].

**Table 1:** Tier 1 genetic tests and conditions.

Condition	Hereditary Breast and Ovarian Cancer (HBOC)	Familial Hypercholeserolemia (FH)	Lynch Syndrome (LS)
Mutations and Inheritance	*BRCA*1/2 mutations; autosomal dominant	*LDLR* mutations most prevalent; autosomal co-dominant	*MLH1*, *MSH2* mutations most prevalent; autosomal dominant
Disease Risk	1%–2% cancer risk per year; lifetime risk—11%–40% for ovarian CA, 40%–80% for breast cancer	Heterozygous: 20-fold increased risk for coronary heart disease (CHD) Homozygous: severe CHD by mid-twenties	80%–85% lifetime risk of colorectal cancer; 20%–60% lifetime risk of endometrial cancer
Type of Testing	*BRCA*1/2 predictive genetic testing	Phenotypic: total cholesterol, LDL-C concentrations Genotypic: DNA sequencing	MSI or IHC tests on tumor tissue, MMR gene mutation testing
Policy Issues	Gaps remain in Medicaid and Medicare coverage of genetic testing	Extremely underdiagnosed (<1% in most countries); controversies in childhood testing	Extremely underdiagnosed (~5% in the U.S.); multiple approaches to screening
Programs	Limited number of state health departments incorporating into surveillance	National FH database initiative starting up	National LS screening Network established

## 2. Methodology: A Consensus Building Approach to Genomics

The *New Strategies* event took place on the Centers for Disease Control and Prevention Roybal Campus on 7 September 2012. The participants, 74 total, came from a variety of sectors: Disease Research and Advocacy (10); Governmental Agency (both CDC and non-CDC health-related) (15); Public Health (state and local public health, public health professional organizations, and university schools of public health) (19); Medicine and Nursing (medical/nursing professional organizations, and university schools of medicine and nursing) (16); Health Care Plans and Systems (8); and Industry (2). Many of the public health and medical/nursing participants had significant genetics-related responsibilities; four individuals worked full-time in genetics but did not fit into any of these categories. Experts in both the clinical and public health domains were present to offer their perspectives and leadership. Industry participants from Myriad Genetics and Genzyme also took part. Members of the “Disease Research and Advocacy” group came from organizations addressing the three Tier 1 conditions—HBOC, LS, and FH—or from agencies that address cancer or heart disease in general. Advocacy representation was important to assure that recommendations would reflect the consumer viewpoint and could secure buy-in from affected individuals and families. Conference goals were for participants to learn about each other’s relevant efforts, foster collaborative relationships, and develop specific recommendations on the implementation of HBOC, LS, and FH initiatives.

**Figure 1:**
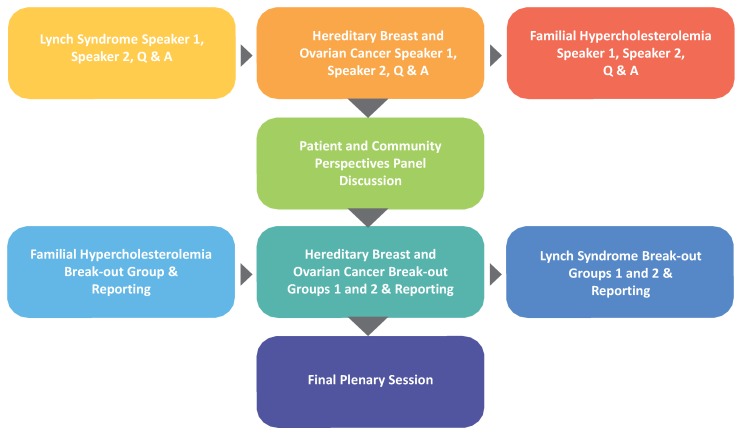
Process used.

Continuity of the event with the preceding CDC-sponsored *Priorities for Public Health Genomics* project was emphasized. The prior project, through the use of the Federal Register, key informant interviews and discussion groups tapped opinion on the expanse of possible directions that public health genomics might take over the next half-decade [12]. The *New Strategies* conference decision-making likewise depended on diverse participant voices. The agenda began with three pairs of speakers, with each disease covered by an expert on the disease condition, followed by an expert on the public health application addressing the disease. A patient and community perspectives panel session followed, which led to working group sessions yielding group recommendations on how to implement each of the three applications (Figure 1). A plenary session concluded with summary reports from the working group session facilitators on the strategies their groups developed.

**Figure 2:**
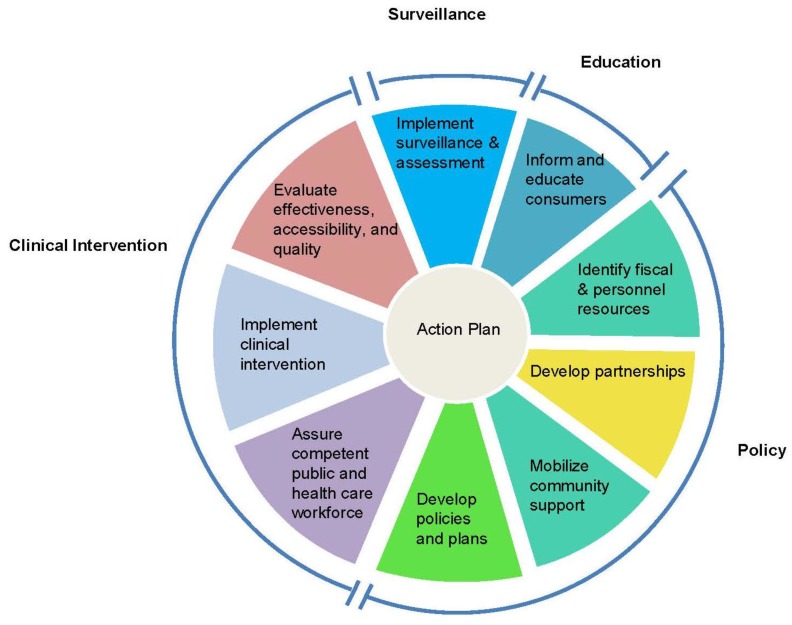
Essential public health functions framework.

Muin Khoury, M.D., Ph.D., director of the CDC-OPHG, introduced an adaptation of the *Public Health in America* framework [13] that the groups could use as a conceptual template for developing an action plan for Tier 1 genetic testing adoption. Originally conceived by the Core Public Health Functions Steering Committee (1994), the adapted essential public health functions framework consists of a wheel with sections devoted to policy development, clinical intervention, surveillance, and consumer education (Figure 2). Inherent in the wheel is an alternation between clinical and public health experts, a balance reflected in the peopling of the conference.

As the conference unfolded, a working group might examine various policymaking strategies to cover the costs of genetic screening, incorporation into existing strategies, or coalition building to enable new program start-up. Discussion of surveillance strategies would lead groups to consider development of surveillance measures and means for patient identification and data sharing, such as use of electronic medical records (EMRs), state health information exchanges (HIEs), and centralized disease registries. Groups were to report on action categories and key features that might constitute a “toolkit” for adoption by state and local public health with relevance to advocacy organizations and professional bodies. An extensive 82-page *Proceedings* (*New Strategies* report; “NSR”), available on the Internet, describes group process and recommendations for the entire conference [14]. Here we examine, for each of the Tier 1 conditions, key implementation recommendations resulting from deliberation within the multidisciplinary groups. These recommendations are summarized in Table 2.

**Table 2:** Select *New Strategies* report recommendations.

**1.** **Collaboration and Partnership Building**
The formation of coalitions and partnerships is critical to implementing screening. Need to include: health care providers, non-profit and patient advocacy organizations, private payers and Medicaid, industry, research (NSR, pp. 29–30).
B. Using advocacy groups that already have large buy-in from public health departments as well as the general public will allow for quicker dispersal of information and testing resources (p. 47).
C. A toolkit for states should include a collection of evidence-based publications, a collection of narratives, and an inventory of successful programs that have been implemented in other states (p. 56).
**2.** **Program Support**
Funding amounts do not necessarily have to be new or large. The action plan may be implemented by partnering with other existing grantees or projects (p. 42).
B. If a patient champion (and possible legislator) is identified, it may be possible for legislation to be enacted on behalf of familial hypercholesterolemia (p. 30).
**3.** **Screening and Surveillance**
In order to create or incorporate these screening programs into public health it is necessary to use preliminary data to document potential saving and cost-effectiveness measures (p. 43).
B. Research and investigation must continue to emphasize the utility of identifying at risk families, covering their counseling visits, and providing HBOC cascade screening to reinforce these clinical practices in order for them to be acknowledged as a standard of care (p. 37).
C. There should be one single FH registry which can be used to find index cases and subsequent cases through cascade testing (p. 27).
D. Universal screening for LS in all confirmed CRC patients should be integrated into the practice of pathology (p. 63).
E. Public health departments should also investigate ways to link relatives of those with CRC to the cancer registries (p. 55).
**4.** **Electronic Health Information**
Utilize state level health information exchange (HIE) to detect potential FH patients, establish a registry system, and monitor health outcomes. Through the registry, there should be state mandated lab reporting of cholesterol over a certain level or threshold through flagging to physicians (p. 27).
B. State health departments should be responsible for developing and distributing quality indicators for HBOC recognition in clinics (p. 45).

## 3. Results

### 3.1. Conference Findings on Hereditary Breast and Ovarian Cancer

#### 3.1.1. Hereditary Breast and Ovarian Cancer: Characteristics and Collaboration

For HBOC, the clinical description was presented by Mark Robson, M.D. of the Memorial Sloan-Kettering Cancer Center, and the public health viewpoint by Amber Roche, M.P.H. from Seattle and King County Public Health. Dr. Robson discussed the epidemiology of the condition—the relative risk for breast cancer given a positive family history, the likelihood of women with breast or ovarian cancer harboring BRCA1/2 mutations, and the risk of carriers in various age groups developing these two cancers. Ms. Roche covered the ideal sequence of BRCA1/2 genetic testing, starting with an index case, assessing the risk status of various family members through the collection of a complete family history, making referrals for genetic counseling, and conducting cascade screening of appropriate relatives. An important take-home message from both speakers was that clinicians and public health practitioners can collaborate in the identification of at-risk individuals and family members.

Community participants—representatives of non-governmental advocacy organizations—provided the “inclusive conscience” for the discussion. The late Rochelle Shoretz of *Sharsheret: Your Jewish Community Facing Breast Cancer* remarked that having the community involved at the very beginning of strategy and policy discussions is important (NSR, p. 16). Participants in the Patient and Community Perspectives Panel agreed that inviting the community to the discussion table when the program is still on the ground floor will build goodwill, collaboration, and mutual respect. Community input in the design phase is also important because community members are the ultimate consumers of genetics services. Sue Friedman of *Facing Our Risk of Cancer Empowered* (FORCE) reminded participants that cost and fear of stigmatization are among the top barriers to acceptance of genetic testing, especially for specific racial and ethnic groups. Panelists expressed concern over lack of consumer and provider awareness of the protections offered by the Genetic Information Nondiscrimination Act (GINA). Due to gaps in Medicaid and Medicare coverage of HBOC testing among those with a positive family history who have never been diagnosed with cancer, and in reimbursement for genetic counseling, financial barriers are much more significant for those depending on these sources for their health care, the Affordable Care Act notwithstanding. The HBOC Working Groups concluded: “In order to achieve the desired policy changes, there is a need for partnerships. The community needs to be engaged in policy-making efforts. Champions in insurance companies need to be involved with the push for coverage expansion” (NSR, pp. 36–37). Voices representing industry can also be quite useful at the table in providing their own experiences with the criteria being used for conducting testing and data concerning testing coverage.

#### 3.1.2. Hereditary Breast and Ovarian Cancer Program Support

In line with the adapted Essential Public Health Functions Framework, the HBOC Working Groups identified funding as a critical element determining the success or failure of Tier 1 screening program implementation. HBOC Group 1 members observed that lack of screening program funding makes it difficult to obtain buy-in at all levels, from individual to institutional. Speaker Amber Roche indicated that a combination of federal (CDC), state, and Komen funding helps low-income uninsured and underinsured clients obtain breast, cervical, and colorectal cancer (CRC) screenings in her state. Surveillance for HBOC incidence and BRCA1/2 genetic testing utilization is also a public health function in several states [15]. The necessary data sharing and education—of the lay community, health care providers, and policy makers—depend on funding. Public understanding of need is an essential driver, as it can lead to recruitment of the right people and engage advocacy organizations, which can provide financial and organizational support to enable larger scale initiatives towards change. Group members advocated for the formation of a “multidisciplinary team of HBOC screening champions” who could raise awareness through the consumer and health care communities.

HBOC Group 2 members concluded that funding does not need to be either new or extensive to achieve program goals. Examples were supplied showing that specific action steps may be accomplished by partnering with existing grantees and projects, some efforts using money from small grants to cover the initial costs.

#### 3.1.3. Hereditary Breast and Ovarian Cancer Screening Practice

For all three conditions, conference participants advocated linking Tier 1 genetic counseling and testing with other ongoing interventions when possible, e.g., HBOC family history assessment and genetic testing with general breast cancer screening; FH screening with existing cholesterol screening protocols; and IHC and MSI screening for LS genetic testing with general CRC screening (NSR, pp. 9, 25, 60). Healthy People 2020 has as an objective a 10% improvement in the proportion of women with a family history of HBOC who receive genetic counseling [16]. HBOC Group 1 commented: “We need a process to measure the efficacy of genetic counseling. Health care providers and public health practitioners, administrators and members of the public need to better understand and acknowledge the value of genetic counseling” (NSR, p. 36). Research and investigation must continue to emphasize the utility of identifying high risk families, covering their counseling visits, and providing HBOC cascade screening to reinforce these clinical practices in order for them to be acknowledged as a standard of care. Group 2 indicated the need to document the cost savings of including HBOC counseling and testing in public health programs.

Given that the U.S. Surgeon General’s Office, CDC-OPHG and advocacy organizations have pressed for the usage of family health history, not just individualized medical histories, groups advised that “a family history intake should become a standard part of care, and should be further recorded and tracked via [electronic] medical records across the population” (NSR, p. 38). Amber Roche described Washington state’s incorporation of family history into its current public health breast cancer screening and detection programs, and clarified that two options exist for integrating family history into breast cancer screening programs: (1) the focused approach described earlier in which public health departments start with women who have been diagnosed with a BRCA1/2 mutation then move on to family members; and (2) a broad approach involving reviewing the records of all clients in a state’s breast, cervical, and colon cancer program, and asking providers to identify patients within their practice at significant risk of carrying an HBOC mutation. Public health practitioners are quite familiar with STD/HIV contact tracing involving direct contact of those potentially affected, but in the genetics realm, family contact through the proband is a viable alternative. HBOC Workgroup members advocated relaying information about the availability of cascade screening along with a proband’s positive genetic test during appropriate genetic counseling, while also recognizing that some family members may be reluctant to share cancer family history (NSR, pp. 36, 40). Public health administrators need to find out what information (cancer family history, clinical, or public health genetic testing data) is being collected by their state cancer registries to utilize this information for state reporting. Using current clinical tools and protocols, providers should be willing and able to enter appropriate patient data for state surveillance use. Indeed, Michigan, Minnesota, and Utah have already taken advantage of existing clinical data—cancer family histories—by accessing doctors’ office chart reviews, electronic medical records, and information from local public health encounters [17]. Conversely, state health departments could help develop quality indicators for HBOC recognition for use in community and health system clinics.

### 3.2. Conference Findings on Familial Hypercholesterolemia

#### 3.2.1. Familial Hypercholesterolemia: Characteristics and Collaboration

James Underberg, M.D., M.S. of the New York University School of Medicine and NYU Center for Prevention of Cardiovascular Disease, who offered the clinical perspective, pointed out that heterozygous FH is just as common a condition as Type 1 diabetes. Patient identification is important—at least 1/500 Americans are thought to be affected by FH, though among these individuals probably only 2% are identified and given the opportunity to be treated more rigorously and earlier than those with “garden-variety” high cholesterol. Katherine Wilemon, founder and president of the *FH Foundation*, reminded those in attendance of the need for education, both in the medical community and within the public conversation (NSR, p. 18). Joan Ware, B.S.N., M.P.H. of Utah’s Family High Risk Program discussed the various tools available for impacting the health of multiple generations of family members—collection of family health history up to three generations deep, the creation of a family health history registry, and data collection via electronic health records to improve surveillance of those at-risk.

The Utah program has identified 8546 high risk families. The key, she stated, was “collaboration and partnerships across stakeholder groups to identify the approximately 600,000 Americans with FH who have not been identified” (NSR, p. 13). One lesson learned is that treating families is usually more effective than treating individuals. Ware also made a series of practical suggestions—that emphasis should be placed on: (1) funding efforts; (2) seeking program “champions”; and (3) securing provider acceptance. Members later in the conference listed the different categories of stakeholders necessary to implement cascade screening of relatives and pediatric cholesterol screening.

#### 3.2.2. Familial Hypercholesterolemia Program Support

Participants distinguished between coverage of individualized testing and support for screening programs. In Britain and the Netherlands, FH cascade screening is supported either by the state or the program itself if it is investigational [18,19]. Likewise, newborn screening in the U.S. is heavily subsidized, with any residual costs picked up by the family's payer. In the case of FH screening within the U.S., which can be performed either through cholesterol screening or mutational testing, the remarks of the group were predictive of current health care reforms. Working group members argued that medical payers would need to be among the partners in efforts by public health and health care providers to implement the screening programs. Private payers and Medicaid would need to support systematic screening, especially if many children are to be involved. Participants reasoned that if a patient champion or engaged legislator could be identified, then it might be possible for legislation to be enacted on behalf of FH. Federal funding, written into larger cooperative agreements, would be useful for the formation of task forces for FH screening, just as it has been with newborn screening. This set of conclusions took a different tack from that reached by the HBOC Working Groups, which advocated incremental funding. The public is generally aware of the dilemma of hypercholesterolemia; that elevated cholesterol is a prevalent condition; and that medications are available to treat this condition. These factors suggest readiness for concerted efforts and the prospect of large-scale funding of FH Tier 1 applications through public and private sources.

#### 3.2.3. Familial Hypercholesterolemia Screening Practice

A number of approaches exist for screening FH in pediatric and adult populations—targeted cholesterol and/or genetic screening based on risk factors or symptoms, cascade screening of relatives, and universal screening of children [20,21]. In the Netherlands, clinical criteria including elevated cholesterol and a personal and family history of coronary artery disease can lead to genetic testing, and entry into a registry for contact of other family members [22]. Cascade screening literature from the United Kingdom and Scandinavia shows a great deal of sensitivity towards contact of family members [19] and, indeed, FH Workgroup participants advised “educating physicians and patients on a broader scale about how to communicate with family members” (NSR, p. 29). Participants recommended creating cascade screening projects based on existing international programs. Direct contact by physician *versus* indirect contact by family members, the more frequent strategy in the U.S. for genetic conditions, is a balance between protection of privacy and the right not-to-know, and accuracy and efficiency of information reaching at-risk relatives [19]. Professional organizations have touted universal screening of children via lipid profiles [23]. Genetic testing in children appears reserved for instances where family work-up has already identified a responsible mutation [24,25], and in adults where LDL-C has reached a critical threshold [26]. FH Workgroup members indicated there should be state-mandated lab reporting of cholesterol over a certain threshold through flagging to physicians.

It is clear that for maximal effectiveness, FH screening within families should start at an early age. As James Underberg pointed out, studies in the Netherlands have demonstrated that cascade screening of family members can lead to effective follow-up, *i.e.*, an increase in cholesterol-lowering treatment [27]. Intervening during childhood (targeted screening after age 2; universal screening starting at 9) can prevent cardiovascular events in adulthood, and even earlier for those with a predisposition to FH. However, he also pointed out that early diagnosis has garnered controversy. Concerns range from the possibility of missed diagnoses to long-term effects of medication, anxiety, and a lack of rigorous outcome studies; thus the need for continued discussion about risks and benefits.

Participants agreed on the need for more far-sighted population monitoring, the international Make Early Diagnosis to Prevent Early Death (MEDPED) FH Registry, with data analyzed at the University of Utah, being one example [28]. The FH Working Group suggested the creation of a national FH registry through collaboration between health system administrators and the U.S. Office of the National Coordinator for Health. It was also suggested that early detection could begin in the schools, as has been done with high school students and their families in Utah [29].

### 3.3. Conference Findings on Lynch Syndrome

#### 3.3.1. Lynch Syndrome: Characteristics and Collaboration

Heather Hampel, M.S., C.G.C. of Ohio State University shared the clinical features and epidemiology of Lynch syndrome. LS is the most common heritable cause of colorectal and endometrial cancer (1/35 CRC and 1/40 EC patients, respectively). If a presymptomatic individual identified with an LS-causing mutation undergoes colonoscopies starting at the recommended age and at the recommended frequency, CRC incidence and related mortality can be significantly reduced. Lives can be saved by diagnosing it early, and universal screening for LS among newly diagnosed CRC patients has been found feasible and cost-effective. Debra Duquette, M.S., C.G.C. of the Michigan Department of Health and Human Services reported that the vast majority of families with a history of CRC do not know they may have LS, or even that a genetic test is available. Duquette emphasized the value of multidisciplinary effort to promote universal LS screening on all newly diagnosed colorectal tumors. “LS is a genetic condition that brings clinical, public health, and advocacy groups together. It may be used as a model of how to build local, regional, national, and international collaboration” (NSR, p. 5). Both individual and institutional buy-in are needed. Participants suggested that individuals and families can promote screening through sharing real life stories that can resonate with all and reduce stigmatization of those affected.

#### 3.3.2. Lynch Syndrome Program Support

LS Group 1 suggested that the CDC could foster collaboration by creating requests for proposals (RFPs) necessitating that public health departments and advocacy groups work together towards a population-based response. It could be that such efforts would require linkage between the two U.S. federal health agencies—CDC and the National Institutes of Health (NIH)—to ensure adequate funding for relevant activities in numerous states. The earlier Priorities Report suggested that new or bolstered genetic screening programs could piggyback on existing funded programs, such as CDC’s Colorectal Cancer Control Program and its National Breast and Cervical Cancer Early Detection Program [12]. This solution parallels LS Working Group 1’s suggestion that the CDC could bring on-board the state Comprehensive Cancer programs [30]. The piggybacking idea has the advantage of prior evidence of success and cost-effectiveness from state programs that have been up-and-running, which participants viewed as important to the startup of new programs. CDC’s Colorectal Cancer Control Program currently provides funding to 25 states for screening programs that promote population-based CRC screening and follow-up for underinsured or uninsured individuals ages 50–64 [31], and its National Breast and Cervical Cancer Early Detection Program supports screening services to low-income, uninsured or underinsured women in all 50 states [32]. If a central body such as the CDC could bring key state examples to bear on the implementation of Tier 1 genetic screening and coordinate agencies that have been involved in the process, then such a “knowledge network” could help train other states. LS Group 1 members envisioned a toolkit for states including a list of evidence–based publications, a collection of narratives, and an inventory of successful programs that have been implemented in other states. States with approved coverage for Tier 1 testing could create active partnerships with other states engaging in Medicaid expansion or willing to build screening programs around tests that are already covered. Coordinated effort is needed by health care plans as well as the genetic testing industry for Medicaid and Medicare to support LS cascade screening, e.g., through the DRG amounts that are supplied to hospitals. LS Group 2 cited advocacy groups as well as the media as conduits for working with communities and publicizing programs at their start-up. Partnering with advocacy groups that already have large buy-in from public health departments could be profitable [12,14]. These relationships would allow the identification of at-risk families and make cascade screening more efficient and less costly.

#### 3.3.3. Lynch Syndrome Screening Practice

A CDC multidisciplinary working group estimated that approximately 4200 cases of Lynch syndrome could be identified each year through routine LS genetic screening on all cases of colorectal cancer in the U.S. [33]. An additional 1000 LS cases could be identified through screening all newly diagnosed endometrial cancers. The working group concluded that using a public health approach strongly integrated with all aspects of clinical care might provide the greatest opportunity for successful implementation on a wide scale. LS Group 1 pointed out that for such screening to be truly “universal”, programs would need to be expanded to allow new EC cases to be included as well. This expansion should be integrated into the practice of pathology laboratories. Family members can also benefit once a proband is tested and found positive [34]. Depending on the particular context, genetic testing for LS can start with the relatives of a patient who has tested positive for Lynch syndrome, or with all the patients in a given clinical setting who have been newly diagnosed with CRC independent of the existence of a related proband with LS. A cultural factor also exists. Investigators have found varied levels of acceptance by different racial-ethnic groups of screening for colorectal cancer, both due to the technique being used and the fear of being at risk for a familial cancer [15,35]. Participants endorsed “review of existing literature on culturally specific factors relating to seeking and avoiding genetic testing in various population groups” (NSR, p. 65).

Engagement of the public health system would entail scanning records within existing state cancer registries for instances of LS testing. LS Group 2 advised, “Data should be collected through cancer registries on all measures of universal screening for Lynch syndrome”, from immunohistochemical means to mutation testing (NSR, pp. 63–64). Group 1 concluded that a centralized database to track compliance and outcomes needs to be developed to improve surveillance and inform outcome measures. Public health departments should also investigate ways to link relatives of those with CRC to the registries.

### 3.4. Use of Electronic Medical Records

Each of the groups advocated the use of electronic medical records (EMRs) to streamline information collection, improve case finding, and identify at-risk relatives who should be offered genetic counseling and testing. Indeed, the forerunning Priorities conference had as an action step the integration of electronic health records to improve the coordination of care and assist in decision support (PR, pp. 38, 55). Health practitioners and advocacy groups are in a position to promote the use of an electronic version of family health history among providers and patients. This objective would carry on the mission of the Surgeon General’s Office and its Family Health Portrait Tool [36]. Recommendations addressed how this vision might be accomplished, including linkage with death certificates via EMRs and establishment of secondary data systems beyond index cases to inform providers of at-risk family members. A standardized tool within EMR systems would need to be developed for provider use to flag individuals who should be made aware of their risk status and available options.

## 4. Discussion

### 4.1. Current State of Tier 1 Genetic Testing and Project Tie-In

A review of recent clinical practice guidelines and literature on cascade screening, universal screening, and genetic surveillance programs suggests that progress is taking place for all three Tier 1 conditions (Table 3). Dialogue is needed to institute and refine such programs. The *New Strategies* conference presaged some developments, and identified other objectives which are yet to be fulfilled.

#### 4.1.1. Hereditary Breast and Ovarian Cancer Update and Identified Gaps

Multiple professional organizations have since this conference was held produced recommendations on the offering of BRCA1/2 mutation testing in conjunction with counseling and the use of family history [37,38]. In the most recent U.S. Preventive Services Task Force (USPSTF) cancer risk assessment guidelines, women with positive preliminary screens using one of several designated screening tools, and family members with breast, ovarian, tubal, or peritoneal cancer should move on to genetic counseling, and if indicated, BRCA genetic testing [37]. Community engagement and advocacy organization involvement will be important ingredients in overcoming limits that efforts to expand the reach of HBOC genetic testing and counseling will encounter.

Currently a handful of states maintain cancer registries that collect data on numbers of persons being given HBOC genetic counseling and testing [39,40]. The possibility of utilizing HBOC screening as a prevention tool on a broader scale than is now occurring has been investigated. Mary-Claire King, Efrat Gabai-Kapara and colleagues in 2014 conducted a population-based BRCA1/2 screening program in Israel starting with 8000 healthy Ashkenazi men and leading to the identification of 211 female carrier family members (youngest age 20; median age 56) [41]. King envisions genetic testing for breast and ovarian cancer mutations of every woman at about age 30 in the course of routine medical care, independent of Ashkenzic ancestry [42]. This practice trajectory is part of the ongoing discussion that needs to take place. For such an eventuality, ongoing evaluation of genetic counseling and testing follow-up is needed, and collaboration between advocacy groups and professional organizations needs to be energized to the extent that elected officials and state health departments are swayed (NSR, p. 43). In the present, a disparity exists in the proportion of at-risk individuals receiving BRCA testing in comprehensive cancer centers and cancer genetics clinics (~70%), and the proportion receiving testing as reflected in medical claims and administrative information from databases of privately insured individuals (~30%) [43]. Yet, Myriad Genetics data suggest that two-thirds of the test orders in Washington state are coming from primary care offices. These differentials speak to the importance of consumer and provider education as well as the multi-source state cancer surveillance programs on which participants commented.

Participants advised, “Be sure that all clinicians are aware and educated on the USPSTF recommendations regarding HBOC” (NSR, p. 45). Many states offer packages, from educational modules to webinars, which address provider HBOC educational needs [40]. The Connecticut Department of Public Health is piloting placement of genetic counselors with primary care practitioners to provide ongoing education and case consultation for BRCA-related cancer. A collaboration between the American Public Health Association Genomics Forum and Genetic Alliance has led to the production of webinars for the three Tier 1 conditions that have been attended by public health practitioners across the nation.

#### 4.1.2. Familial Hypercholesterolemia Update and Identified Gaps

Testing for familial hypercholesterolemia is supported by National Institute for Health and Clinical Excellence (NICE; U.K.) and USPSTF guidelines, both of which emerged in 2008 [6,44]. The NICE guidelines speak to the value of both phenotypic (cholesterol levels) and genotypic (mutational) means of diagnosis, and advocate “a nationwide, family-based, follow-up system” for cascade testing, including at least first- and second-degree relatives [6]. Participants noted private payers in some states beginning to cover lipid screening as young as age 2 and for teens. U.S. National Lipid Association Guidelines recommend universal screening for elevated LDL in those less than 20, while the National Heart Lung, and Blood Institute and American Academy of Pediatrics advocate lipid screening on all children between the ages of 9 and 11, and 17 and 21 [23,24,25].

While the U.S. has created policy for universal cholesterol screening of adults (USPSTF) and children (NHLBI, APA), implementation has been slow. Several European countries are already systematically enrolling children in cholesterol screening in the schools and the clinics [23,44,45], and engaging family members in genetic screening for FH [46]. Recent cascade screening efforts have benefited from two developments—centralization of data on families and children tested [47], and increased availability of highly effective statins and other drugs [48,49]. The FH Workgroup indicated that “a diagnosis of FH should automatically allow for coverage of appropriate medications and prescriptions, as indicated by the NICE guidelines” (NSR, p. 25). Recent developments parallel the FH Group recommendation for a national FH registry through a coalition of different sectors and public health organizations. In 2013 the non-profit FH Foundation launched the CASCADE FH Registry, which contains both clinically-based and population-based data on individuals with FH [50]. The Registry is actively tracking information on patients with a family history of FH, on those who have been diagnosed with the condition, and on those who are now taking cholesterol-lowering medications [46,51]. In 2015 three groups—the FH Foundation, Stanford Medicine, and Amgen—launched the *Find FH* Initiative through funding from the American Heart Association and Amgen. The program will use computer-based algorithms to mine large databases including EMRs in order to identify patients likely to have FH [52]. The FH Workgroup argued for utilizing EMRs for this purpose and to flag patients through physician notification.

#### 4.1.3. Lynch Syndrome Update and Identified Gaps

In 2015 NCCN published clinical practice guidelines distinct to genetic/familial high-risk assessment of colorectal cancer, including Lynch syndrome [53]. Very much in line with conference recommendations, NCCN proposed that newly diagnosed CRC patients be screened for Lynch syndrome. The EGAPP Working Group found sufficient evidence to recommend genetic testing for individuals with newly diagnosed colorectal cancer, but insufficient evidence to recommend choice of a specific molecular screening and mutation testing strategy [54]. A 2014 consensus statement by the US Multi-Society Task Force on Colorectal Cancer notes that among the options examined by EGAPP, a strategy involving initial IHC testing has proven the most cost-effective [55].

In a clinical cancer LS surveillance program in Finland, 75% of high-risk members of families with identified LS mutations received counseling and ultimately were tested [56]. Health systems such as Kaiser Permanente Northwest have found a general level of acceptance of the possibility of undergoing LS testing among their CRC positive enrollees [57]. However, a review by Sharaf *et al.* indicates that LS genetic testing among first degree relatives remains underutilized [58]. Bellcross *et al.* have cautioned that universal LS screening “will require significant collaborations between healthcare systems and public health agencies, with strategies and interventions targeted at multiple levels across the healthcare continuum” [33]. This comment echoes the perceived need for partnership building and networking to make LS universal screening work, as discussed at the conference. The Lynch Syndrome Screening Network, comprised of 66 institutions, reports that the majority of its member institutions have implemented universal screening practices, an indicator that universal screening is feasible for motivated organizations [59]. Established with funding by the CDC-OPHG, this network could benefit from continued collaboration with the CDC through its Colorectal Cancer Control Program to widen access to LS genetic testing to the uninsured and underinsured in the Program’s member states.

#### 4.1.4. Emergent Electronic Networks and Health Care Reform

Conference participants were enthusiastic about the potential usefulness of electronic medical records, which can greatly assist public health surveillance efforts. A number of states have formed electronic health information networks that place health departments at the hub of the health information exchanges that are forming. Michigan and Minnesota, for example, have systems that allow the sending and receiving of clinical information between providers and health care organizations, and of health information between medical systems and health departments [60,61]. A 2012 Association of State and Territorial Health Officials (ASTHO) report describes various state efforts, including those in New York, Rhode Island, Indiana, and Utah, to create comprehensive electronic systems that electronically link infectious disease, immunization record, newborn screening, and birth defects tracking [62]. These systems are states’ electronic answers to the health care exchanges required under the Patient Protection and Affordable Care Act (ACA), building on the momentum of prior legislation such as the Health Insurance Portability and Accountability Act (HIPAA) which paved the way for secure sharing of electronic health records. This progress within the U.S. system is a step in the right direction for individuals and families who might benefit from Tier 1 applications, such as the BRCA genetic counseling listed in the ACA’s list of covered preventive services, and respective surveillance systems [63].

**Table 3:** Tier 1 Genetic testing—Current progress.

	Policy Guidelines			Cascade Screening		Universal Screening		Surveillance and Registries	
Condition	Published (Y/N)	Implemented (Y/N)	References ^a^	Achieved (Y/N) ^b^	References	Achieved (Y/N)	References	Achieved (Y/N)	References
Hereditary Breast and Ovarian Cancer (HBOC)	Y	Y	USPSTF [37]; NCCN [38]; HHS.gov [63]	N	George *et al.* [4]; Cody *et al.* [64]	N	Gabai-Kapara *et al.* [41]	(Y) ^c^	Katapodi *et al.* [39]; CDC [40]
Familial Hypercholest-erolemia (FH)	Y	(Y)	NICE [6]; NHLBI [23]; Knowles *et al.* [26]; USPSTF [44]	N	Langslet and Ose [18]; Neal *et al.* [46]; Avis *et al.* [47]; Bell *et al.* [48]	(Y)	Peterson *et al.* [20]; Sullivan *et al.* [21]; Kusters *et al.* [45]	Y	Williams *et al.* [28]; Neal *et al.* [46]; O’Brien *et al.* [51]; Stanford Medicine [52]
Lynch Syndrome (LS)	Y	(Y)	NCCN [53]; EGAPP [54]; Giardiello *et al.* [55]	(Y)	Hampel *et al.* [34]; Dilzell *et al.* [65]; Atkan-Collan *et al.* [56]; Sharaf *et al.* [58]	(Y)	Bellcross *et al.* [33]; Schneider *et al.* [66]; Hunter *et al.* [57]	Y	Mange *et al.* [59]

^a^ Citation numbers appear according to their location in the text; ^b^ Cell will be marked “Y” only if program is occurring in both Europe and the U.S.A; ^c^ “(Y)” means limited achievement of the policy or program at the current time.

### 4.2. Implications and Lessons Learned

Practice guidelines (USPSTF, NCCN) are now shifting to include family history and “high-risk assessment” for heritable cancer conditions in their recommendations. This conference outlined areas where the building of clinical evidence and offering of professional genetics education need to continue for the guidelines to be fully implemented and for Tier 1 genetic testing to be conducted more broadly. A state of readiness naturally exists in state health departments containing staff devoted to the offering and monitoring of validated genetic testing. Genetic interventions fight with other competing needs on the public health front, thus the value of collaborations to mount any widespread effort at cascade screening of family members, or universal screening of those at-risk for the Tier 1 conditions. These collaborations need to be multi-sectoral. Public health surveillance of genetic conditions depends in part on clinical case reporting. Physicians can benefit from 2-way reporting, both in terms of aggregate statistics and alerts pertaining to at-risk family members. Future interconnections between the medical and public health workforces will be even more intimately entrenched as EMRs and HIEs become widely used. Industry will be part of the collaboration, first through its attention to the coverage of genetic tests and specific criteria for testing, then through its participation in burgeoning networks like the Lynch Syndrome Screening Network.

Tier 1 genetic programs also depend for their inception on decision-making processes involving health professionals and policy makers. Major decisions need to be made if LS genetic testing is to be offered to all CRC patients, and if HBOC genetic screening is to be offered to women starting age 30 in the course of routine medical care. It is not surprising that consensus conferences have been at the heart of Lynch syndrome policy making over the last four years [33,55]. Collective deliberation allows for the promotion of mutually respectful decision-making, and furthers the legitimacy of collective decisions [67]. The first feature allows for continued discussion that can isolate areas of disagreement and resolve difficult issues, ultimately yielding to deliberation. The second feature is an important ingredient of buy-in by community groups. Gutmann and Thompson [67], and Yankelovich [68] have noted the importance of including both expert and lay opinion in social and healthcare decision making. The *New Strategies in Public Health Genomics:* *Actions to Save Lives Now* conference, given its constituency break-down, offers a good example of a deliberative setting comprised of representative members who will be implementing and those who will be affected by genetic screening programs.

The deepest message of the conference was that cooperation and collaboration are necessary for real change to come about. Policy proposing programmatic change, no matter how detailed, can meet significant resistance if it does not have a willing constituency and material backing. This conference showed that health professionals and health advocates can develop realistic recommendations when they combine their expertise. Conference-goers learned the value of bringing a diverse group of people together. This lesson is especially important given the bridgeable divide that has existed in the past between the medical and public health communities. Community advocates were important to the process of discerning what human connections are needed to implement programs in an acceptable and successful way.

The splitting of conference participants into several groups addressing three specific Tier 1 applications proved more productive than focusing on just one condition, and resulted in both convergent and divergent sets of recommendations. Collectively, the different groups make an effective case for implementation of cascade genetic screening and universal screening as indicated. The sequence of recent events suggests that conference recommendations about genetic screening, surveillance programs and network establishment can point the way, and contribute to changes waiting to take place in the genetics arena. Indeed, by March 2014 CDC-OPHG had bundled the various ideas together and released Tier 1 toolkits for state and local public health containing information on relevant registries and registry reporting, surveillance indicators, educational programs and materials, and publications [69].

## 5. Conclusions: A Less Receding Horizon

The above overview of recent developments demonstrates a significant set of activities and body of literature arising since 2012, the year the *New Strategies* conference took place. For the three Tier 1 conditions—HBOC, FH, and LS—testing in Europe has benefited from national health systems and ongoing clinical trials, while in the U.S. commercial health care systems and disease advocacy-related networks have much more recently taken on some of the responsibility. Health care reform in the U.S. could increase availability to many more persons than are currently being counseled or tested. Public health can be a major player in identifying and educating individuals and families who might benefit, and in developing coalitions combining the strengths of clinical medicine, public health practice, and the community at large. The assimilation of data on individuals at-risk and pooling into electronic systems, a goal prioritized by conference participants, is rushing forward fairly rapidly in the U.S. due to HIPAA and Affordable Care Act requirements, and the involvement of multiple sectors. We have hoped to show in this paper a confluence of efforts by federal agencies, state public health departments, disease advocacy organizations, and industry. BRCA genetic testing is now covered under the ACA, and Medicare covers multi-target stool DNA testing for CRC [70,71].

Genetic testing policy remains in evolution. The shift in NHLBI’s policy from screening children with a family history of cardiovascular disease or dyslipidemia to screening all children between the ages of 9 and 11, and 17 and 21 represents progress. A variety of issues remains unresolved, however. One is contact policy in the case of cascade screening. Should it be done routinely by the healthcare team, or by the proband and close family members? Those at risk—affected groups and families—should have a stake, particularly because preferences in the U.S. may differ from those in other countries. Also, from the resource standpoint, should the focus of attention be on cascade screening, which exists at the family level, or universal screening of those meeting established criteria? Can the envisioned “toolkit” to help states interested in initiating these applications be employed in a way that caters to different states’ needs? The conference outlined where actions are needed, but final choices remain, calling for further dialogue.

This conference and the preceding *Priorities for Public Health Genomics* conference share a trickle-down effect. They both invoke opinion from people aware of the trends in the technology, and activate opinion by identifying key points for further action. Consensus building in genetics can have an impact. It needs to continue in the domain of public health genomics to keep the field, not just the people impacted, “healthy.” Professional practice guidelines and state-wide regulations will benefit, since they reflect both incoming factual evidence and public acceptability of new and emerging technologies.

With any dialogic exchange, the timeliness issue acts as a barometer of the potential relevance of what is discussed. The United States has just emerged from an explosion of events in the health care arena, including the liberalization of gene patenting and an overhaul of the national health care system. The U.S. and Europe are closer together health policy-wise than they have been in decades. The price of BRCA1/2 genetic testing is significantly down, and new tests like Cologuard © for colon cancer screening as well as newly approved cholesterol-fighting compounds are coming to market. Truly a new window of opportunity exists. Will the pace of opportunity in genetic testing advance one person or family at a time? The discussion in public health genomics favors an umbrella of prevention addressing those at risk throughout the community, with public health as an active agent and beneficiary in the array of changes visible on the horizon.
